# Anaphylactoid to polyurethane foam (yellow foam) among architects

**DOI:** 10.4103/0972-5229.60177

**Published:** 2009

**Authors:** Viroj Wiwanitkit

**Affiliations:** Wiwanitkit House, Bangkahe, Bangkok, Thailand 10160

Dear Editor,

Polyurethane foam or yellow foam is a widely used substance. Allergy to polyurethane foam is rarely reported. Yellow foam is as an important cause of occupational asthma.[1] Workers in a polyurethane manufacturing foam factory are the main risk group for getting allergy.[2] The presence of isocyanates in polyurethane foams is a possible contributor in asthma.[2] However, there are only a few reports regarding this kind of allergy. We have seen two cases of anaphylactoid reaction to polyurethane foam in students doing architecture, who use polyurethane in their architecture model construction. Both cases (male, 19 years old) studied in the same class, developed sudden respiratory difficulty during the use of polyurethane foam and there were numerous wheals on their skins. These two cases were taken to the emergency care unit where they were found to be hypotensive. Both cases were immediately resuscitated with intravenous fluids and dexamethasone administration and recovered within 15 minutes.

Architects who use polyurethane foam in daily work, can be considered as a high risk group to polyurethane allergy.

## References

[1] Bousová K (2004). Occupational asthma: current state of the problem. Acta Medica (Hradec Kralove) Suppl.

[2] Candura F, Moscato G (1984). Do amines induce occupational asthma in workers manufacturing polyurethane foams?. Br J Ind Med.

